# The “tyranny of distance”: community-based veteran suicide prevention in Guam

**DOI:** 10.3389/fpubh.2025.1469973

**Published:** 2025-07-28

**Authors:** Joanna R. Sells, Kelly A. Stearns-Yoder, Molly E. Penzenik, Nazanin H. Bahraini, Molly J. Sullan, Selena Cruz, Lindsey L. Monteith, Lisa A. Brenner

**Affiliations:** ^1^Rocky Mountain MIRECC for Veteran Suicide Prevention, Aurora, CO, United States; ^2^Department of Physical Medicine and Rehabilitation, University of Colorado Anschutz Medical Campus, Aurora, CO, United States; ^3^Department of Psychiatry, University of Colorado Anschutz Medical Campus, Aurora, CO, United States; ^4^VA Brain Health Coordinating Center, VA Eastern Colorado Health Care System, Aurora, CO, United States

**Keywords:** Guam, Asian, native Hawaiian or other Pacific Islander, Veteran, suicide prevention, community-based, rural

## Abstract

The population of Guam is comprised of a high proportion of Asian and/or Pacific Islander individuals, many of whom have served in the military. Minoritized community membership and military service are associated with increased suicide risk. This mixed-methods study was conducted to increase understanding regarding the community context, factors associated with suicide risk among Guamanians, and implementation barriers and facilitators for a community-based suicide prevention program, Together with Veterans (TWV). For this convergent parallel mixed-methods study, data were collected from twelve individuals. Qualitative data were analyzed via a rapid analysis approach. Themes were organized using a socio-ecological model framework, then integrated with quantitative data. Emergent themes by socio-ecological levels included: intrapersonal (e.g., identities, mental health stigma); social-interpersonal (e.g., perceptions of Veterans); social community (e.g., access to care); social organizational (e.g., hope for raising awareness); social public policy (e.g., part of, yet isolated from, the mainland); and chronosystem (i.e., geopolitical history). Quantitative data yielded convergent and divergent findings. Findings highlight unique risk and protective factors faced by those living in Guam, as well as the utility of structured community-based suicide prevention strategies to make change among those living in island communities that are geographically distant from the mainland.

## 1 Introduction and context

### 1.1 Guam

Guam is an island of 209 square miles located in the Western Pacific; nearly 3,700 miles southwest of Hawaii, the closest United States (US) land mass (1). Most residents of Guam identify as Asian and/or Pacific Islander [AAPI; (2)]; specifically, Chamorro or CHamoru, who are indigenous to the Mariana Islands (3), Chuukese, from Chuuk State in Micronesia (4), Filipino, or from other Micronesian communities. Like in other AAPI communities across the US, suicide rates are elevated and rising in Guam (5–7).

Following more than 300 years of Spanish rule, including integration of Catholicism, Guam came under American control in 1898. Guam was governed by the US until 1941, when it was seized by Japan during World War II, and occupied until Allied forces retook the island in 1944. The island served as a vital air and naval base for the US until the end of World War II, and remains an important strategic location for the US military (8).

Guam has a population of 153,836 (9) and is home to a growing number of US military members and Veterans, due to its strategic military location (8, 10). There are approximately 9,700 service members stationed across several bases (11), with an additional 5,000 Marines expected to arrive following the recent reactivation of a Marine Corps base on the island (12). Guamanians have high rates of military service, with 10.5% of the adult population being Veterans (11,927 people), compared to 6.8% at the national level (13).

### 1.2 Suicide rates

While most Guamanians identify as AAPI, the US Veteran population is comprised of a relatively small percentage of AAPI individuals [i.e., approximately 2%; (14)]. The age- and sex- adjusted suicide rate among Veterans in 2020 was 57% higher than among non-Veteran adults (15). When disaggregated by race, the unadjusted suicide rate among AAPI Veterans was the second highest rate across all racial groups (15), at 30.2 per 100,000 in 2020 (15). Of further concern, the suicide rate among AAPI Veterans increased 167% between 2001 and 2020, a larger increase than among any other racial and ethnic group (15). Another recent analysis of suicide rates among AAPI Veterans also noted high and increasing suicide rates in this population, with suicide rates among those ages 18–34 exceeding those of similarly aged Veterans (16). In addition to elevated and increasing suicide rates among AAPI Veterans, regional differences have been noted (17).

Suicide rates among members of the general population on Guam are also high, with age-adjusted suicide rates of 30.0 and 21.2 per 100,000 in 2020 and 2021 (5). Suicide rates in Guam are further elevated among those who identify as Pacific Islanders, specifically Chuukese or Chamorro, with suicide rates of 50.4 and 23.8, per 100,000, respectively (5). While the overall rate of suicide across the general US has decreased (6), Guam’s rate increased from 2010 to 2020 (5); highlighting the need for increased understanding of factors influencing risk among those living on the island (7).

### 1.3 Veteran community-based suicide prevention

Suicide prevention is a priority area for the VA, as reflected in the VA National Strategy for Preventing Veteran Suicide, which outlines a public health approach (18). Community-based interventions play a pivotal role in reaching Veterans where they live (18), and implementation often requires leveraging multiple partners and methods. For example, current VA community-based efforts include the Governors’ and Mayor’s Challenges, which engage local and state government and community partners to facilitate interagency suicide prevention planning. VA has also prioritized hiring Community Engagement and Partnership Coordinators, who expand on the work of Suicide Prevention Coordinators by partnering with communities to implement evidence-based suicide prevention strategies (19).

### 1.4 Together with Veterans

Initially funded in 2015, TWV is a community-based suicide prevention approach for individuals residing in rural settings. Communities receive funding, technical assistance, and coaching support for 36 months to implement evidence-based suicide prevention strategies [i.e., provide suicide prevention training; enhance primary care suicide prevention; promote connectedness and help seeking; improve communication across Veteran-serving programs; enhance behavioral health suicide prevention; promote lethal means safety; (20)] in a manner consistent with the needs of their communities. Thirty-eight communities across 26 US states and territories have implemented TWV. For more information regarding TWV, see Kreisel et al. (21) and Monteith et al. (22).

### 1.5 Present study

As Guam is unique in many ways, the decision was made to analyze qualitative and quantitative data from this community separately from other TWV communities. Specifically, the present study aimed to increase understanding regarding the community context, and factors associated with suicide risk among those living in Guam, as well as initial considerations regarding barriers and facilitators to implementing TWV. Qualitative themes were organized by socio-ecological model (SEM) framework domains, in which health behaviors are viewed within the context of multiple levels of influence (23, 24). This model has been used to evaluate a wide range of health promotion efforts (24, 25).

## 2 Materials and methods

### 2.1 Participants and procedures

Data collection was approved by local review boards. Three members of the study team traveled to Guam in September 2022. All are women, and had previous experience conducting qualitative interviews. None are part of the Guamanian community or identified as AAPI or Veterans. One-on-one semi-structured interviews were conducted with a purposive sample of 12 individuals, which included members of the Guam TWV team and other community members who were selected by the TWV community’s leadership team and who were colleagues and peers from various community agencies/groups and were familiar with both the community and the work of TWV, and who agreed to be contacted by our interview team. All participants had indirect or direct community roles related to suicide prevention or associated topics (see demographic details in Table 1). Interviews were conducted in private rooms at a public-serving facility. Members of the study team had been professionally introduced to a few interviewees prior to the visit, and additional interviewees were identified via these contacts, or through the TWV coach.

**Table 1:** Demographic characteristics of key informants (*N* = 12).

Characteristic	*N* (%)
Sex assigned at birth	
Male	8 (66.7%)
Female	4 (33.3%)
Gender	
Men	8 (66.7%)
Women	4 (33.3%)
Racial background	
White	2 (16.7%)
Pacific Islander	10 (83.3%)
Do you identify as Hispanic or Latino(a)?	
No	11 (91.7%)
Yes	1 (8.3%)
What is the highest level of education you have completed?	
High school diploma or equivalent	3 (25.0%)
Some college, no degree	1 (8.3%)
Associate’s degree	1 (8.3%)
Bachelor’s degree	3 (25.0%)
Master’s degree	3 (25.0%)
Doctoral degree	1 (8.3%)
What is your current marital/relationship status?	
Married	10 (83.3%)
Divorced/separated	2 (16.7%)
What is your sexual orientation?	
Heterosexual	11 (91.7%)
Bisexual	1 (8.3%)
What is your current employment status?	
Employed full-time	8 (66.7%)
Not employed, seeking employment	2 (16.7%)
Not employed, not seeking employment	1 (8.3%)
Retired from the workforce	1 (8.3%)
Have you ever served in the U. S. Military?	
Yes	8 (66.7%)
No	4 (33.3%)

### 2.2 Data collected

Interviewers employed an interview guide, which included questions about the experience of living in Guam and community members’ perceptions regarding mental health and suicide prevention. Questions were open-ended, and probes were used as needed to clarify responses (see Supplementary material A1). Interviews (durations ranged from 30 to 90 min) were recorded with permission, and transcribed by a professional transcription service. Informants also completed a demographics questionnaire and three quantitative scales collected using REDCap (26). The Communal Mastery Scale assessed individuals’ beliefs in their ability to attain goals secondary to being interconnected with others (27). Possible total scores ranged from 10 to 40, with higher scores indicating higher communal mastery. The Collective Efficacy Scale (adapted) was administered to obtain information regarding individual beliefs about the capacity of their community to achieve specific goals (28). The possible total score range is 5 to 25, with higher scores indicating belief in the capacity of the community to achieve goals. The TWV Implementation Questionnaire (TWV-IQ) includes questions covering five domains [Planning and Implementation (3 questions); Leadership (5 questions); Community Involvement in the Coalition (6 questions); Progress and Outcome (4 questions)], and Overall Approval (1 question); with two additional binary questions regarding TWV impact on the community and sustainment (see Supplementary Table A2).

### 2.3 Data analysis

Consistent with best practices for convergent mixed-methods analyses, quantitative and qualitative data were analyzed separately, then synthesized into meta-inferences in a joint display table. Meta-inferences were identified as congruent when inferences were drawn from qualitative and quantitative data that aligned and discrepant when inferences were drawn from data that were not aligned. Following the analysis, qualitative themes, quantitative results, and congruent and discrepant findings are reported (Table 2). For more information regarding mixed-methods employed, see pages 68–77, Creswell and Clark (29). For quantitative data, mean, standard deviation, range, median, and 25th and 75th percentile values were calculated. Frequencies were tabulated for the two binary questions.

**Table 2:** Joint display table for mixed-methods findings.

Socioecological level	Qualitative theme(s) (See Supplementary Table B)	Quantitative data			Meta-inferences
		Communal mastery scale (CMS)* Mean (SD) Range	Collective efficacy scale (CES)^†^ Mean (SD) Range	Together with Veterans Implementation Questionnaire (TWV-IQ; Key Findings: Overall Domain and by Specific Questions)^§^ (See Supplementary Table A2 for Full Results)	*Congruent (inferences drawn from the qualitative and quantitative data are aligned)* *Discrepant (inferences drawn from the qualitative and quantitative data are not aligned)*
Intrapersonal - Individual Community Member	Identities; Mental Health Stigma and Difficulty Accessing Resources/ Barriers to Access to Treatment -Individual Experiences	32.3 (3.6) 28–40	21.3 (4.8) 9–25	Leadership (C): C2, C3 Community Involvement in the Coalition (D): D1, D4 Progress and Outcome (E): E1, E2, E3, E4	*Congruent:* Per qualitative interview data, stigma and challenges to accessing care may interfere with making change pertaining to issues related to mental health and suicide prevention. Challenges related to identities and stigma noted in interviews may be reflected in the relatively lower scores on the TWV-IQ Community Involvement Domain. *Discrepant:* Overall, CES scores highlight individuals’ sense that, as a community, they can attain specific goals, despite potential challenges regarding “conflicted” identities and values noted in qualitative interviews. This is further bolstered by strengths in Leadership and Progress and Outcome domains noted on the TWV-IQ.
Social – Interpersonal (community members interacting with each other)	Perceptions of Military/Veterans	---	21.3 (4.8) 9–25	Progress and Outcome (E): E1, E2, E3, E4	*Congruent:* Interviewees’ sense that “everyone wants to help the military and veteran community” appears aligned with both CES and TWV-IQ Progress and Outcome scores.
Social - Community (community member interacting with organization)	Access to Care – Individual Experiences Interacting with Healthcare System; Mental Health Stigma and Difficulty Accessing Resources/ Barriers to Access to Treatment	32.3 (3.6) 28–40	---	Community Involvement in the Coalition (D): D1, D4	*Congruent:* Individuals’ sense about their ability to make change (CMS) is likely related to the persistent nature of healthcare challenges and stigma noted (interviews); as well as challenges engaging key members of their community (TWV-IQ). *Discrepant:* Persistent challenges related to obtaining health care and mental health care, in particular, were noted throughout interviews. Nonetheless, on the CES, members of this TWV community reported the ability to make changes within their community.
Social - Organizational (organizations interacting with each other)	Reasons for Adoption; Hope for Raising Awareness; Barriers and Facilitators	---	21.3 (4.8) 9–25	Planning and Implementation (B): B1, B2, B3 Leadership (C): C1, C2 Progress and Outcome (E): E1, E2, E3, E4 Overall Suggestions and Approval Rating: F, G	*Congruent:* Qualitative data regarding the TWV program was congruent with both CES and TWV-IQ scores (multiple domains and responses).
Social - Public Policy and Federal Systems (national systems of care)	Part of, yet Isolated from the Mainland U. S.; Access to Care – Federal Systems	32.3 (3.6) 28–40	21.3 (4.8) 9–25	Planning and Implementation (B): B1, B2, B3 Leadership (C): C1, C2 Progress and Outcome (E): E1, E2, E3, D4 Overall Suggestions and Approval Rating: F, G	*Congruent:* Significant barriers related to isolation and lack of access noted in interviews are congruent with lower CMS scores. *Discrepant:* Despite significant challenges (noted in interviews), members of this community believe that they can make changes related to suicide prevention (CES) and report success in doing so on TWV-IQ scores (multiple domains and responses).
Chronosystem (change over time)	Geopolitical and Colonial History	32.3 (3.6) 28–40	21.3 (4.8) 9–25	Planning and Implementation (B): B1, B2, B3 Leadership (C): C1, C2 Progress and Outcome (E): E1, E2, E3, E4 Overall Suggestions and Approval Rating: F, G	*Discrepant:* Despite significant challenges noted in interviews: e.g., (“equal in war, but not equal in peace”) members of this community believe that they can make changes related to suicide prevention (CES) and report success in doing so based on TWV-IQ scores (multiple domains and responses).

*CMS: Total Score; 10 item-scale; possible score range 10–40; higher scores indicate higher communal mastery; †CES: Adapted 5-item scale; item score range 5–25, higher scores indicate belief in capacity of community to attain goals. §TWV-IQ: Developed to obtain information regarding TWV program: Domains include: Planning and Implementation, Leadership, Community Involvement; Progress and Outcome, and Overall Suggestions and Approval.

**Figure 1:**
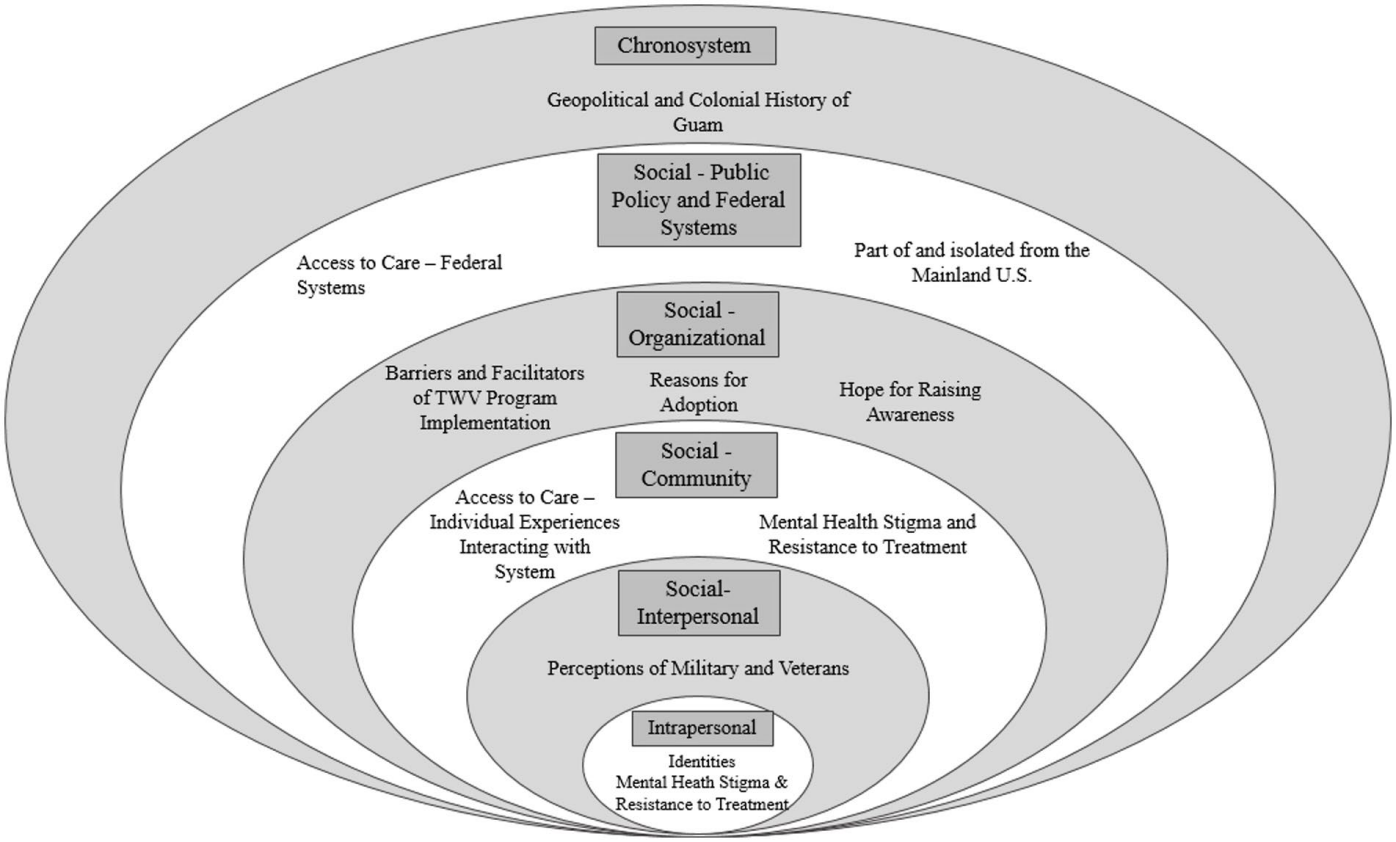
Sociological levels and themes. This figure contains levels including: Intrapersonal: Individual community member. Social-interpersonal: Community members interacting with each other. Social-community: Community members interacting with organization. Social-organizations: Organizations interacting with each other. Social-public policy and federal systems: National systems of care. Chronosystem: Change over time.

Qualitative interviews were analyzed using an approach developed for health services efforts to obtain actionable, targeted data over a shorter period of time than usually required to process such information (30, 31). All four female analysts, none of whom were Guamanian or identified as AAPI or Veterans, had attained a master’s level education or higher and underwent training in qualitative methods. Each interview was analyzed independently by two individuals. Analysts first organized interview content by questions from the interview guide, extracting quotes and summarizing information into a summary template using neutral domain names and a designated section for additional observations. Each analyst independently reviewed a set of transcripts and associated audio recordings as primary or secondary reviewers and summarized information using the template. The team regularly met during analysis to review emergent themes, ensuring investigator triangulation (32). Themes were derived consensually from interview summaries, and emblematic quotes were selected across interviews. A matrix (33) was then used to further organize themes by SEM framework domains (25, 51) including: intrapersonal; social-interpersonal; social-community; social organizational; social-public policy and federal systems; and chronosystem (see Table 2; Figure 1). More specifically, within the SEM, health behaviors are viewed within the context of multiple levels of influence (24) [e.g., individual behaviors, characteristics of the environment (23). The model has been used to evaluate a wide range of health promotion efforts (24, 25)].

## 3 Results

### 3.1 Quantitative data

Demographic characteristics for the 12 interviewees are presented in Table 1. Two-thirds (66.7%) of interviewees were male. The majority identified as Pacific Islander (83.3%), and most (66.7%) had served in the US military.

Results [mean (standard deviation)] from surveys were as follows: Communal Mastery Scale, 32.3 [3.6], and Collective Efficacy Scale 21.3 [4.8] (Table 2). TWV-IQ Planning and Implementation, Leadership, and Progress and Outcome domain scores each exceeded 4 on a 5-point scale. Items regarding satisfaction with community involvement were rated the lowest, with a mean of 3.8 [0.8] points (see Table 2; Supplementary Table A2).

### 3.2 Qualitative data

#### 3.2.1 Intrapersonal-individual (community member)

Two themes emerged within the intrapersonal-individual sphere: (1) internal conflicts experienced by those who identified as both being from Guam and having served in the US military; and (2) internalized mental health stigma and beliefs about seeking treatment. Specifically, interviewees noted shared beliefs and norms between military and Guamanian cultures, which may deter mental health help-seeking behaviors (see Supplementary Table B for illustrative quotes).

#### 3.2.2 Social-interpersonal

This second sphere contained a single theme: (1) perceptions of military servicemembers and Veterans in Guam. Interviewees shared that military service is common among Guamanians and, due to the interconnectedness of the community, most residents know at least one Veteran. Military service is often multigenerational, and Veterans tend to be viewed with pride and respect by the community.

#### 3.2.3 Social-community (community member interacting with organization)

The third sphere included two themes: (1) access to care. Interviewees described frustration Guamanian Veterans experienced when accessing care on the island due to systemic barriers, such as lack of specialty care and residential treatment programs, difficulty navigating care systems, and high provider turnover; and (2) mental health stigma. Interviewees highlighted concerns that seeking mental health care could negatively impact careers. They also noted the prevalence of Catholicism on the island and cited religious beliefs as a contributing factor for stigma around mental health and suicide. Interviewees underscored that mental health stigma was not unique to Guam, but representative of a larger societal phenomenon. Nonetheless, they posited that effective mental health treatment needs to be informed by and responsive to Chamorro culture.

#### 3.2.4 Social-organizational (organization to organization)

Three themes related to the TWV program were noted: (1) reasons for adopting the TWV program; (2) hope for raising awareness regarding suicide prevention and, (3) barriers and facilitators to implementing the TWV program. Interviewees described the community’s desire to foster additional connections with organizations, businesses, and community healthcare. Several interviewees noted the disconnected nature of Veteran services on the island and shared their vision for building partnerships into a web of support for Veterans living in Guam.

#### 3.2.5 Social-public policy and federal systems

Two themes emerged in the Social-Public Policy sphere: (1) dissonance between being part of and separate from the US. In talking about their experiences living in Guam, interviewees contrasted the island’s patriotism and pride in military service with the reality of geographical and cultural isolation from the mainland; and (2) difficulty accessing federal systems of care, including mistrust of government agencies, particularly VA and Department of Defense, as barriers to suicide prevention.

#### 3.2.6 Chronosystem

The Chronosystem encompassed historical events, shifts in sovereign power, and cultural values that have shaped the development of Guam as a community. Interviewees reflected on the impact of their island’s history, culture, and relationship with the US government as part of their experience living in Guam.

### 3.3 Synthesized data

Meta-inferences drawn from the synthesis of quantitative and qualitative results revealed areas of congruence (inferences drawn from the qualitative and quantitative data are aligned) and discrepancy (inferences drawn from the qualitative and quantitative data are not aligned). Information regarding specific data (qualitative, quantitative) by SEM domains used to make such inferences are presented in Table 2 (Joint Display Table for Mixed-Methods Findings). Overall, results indicated that despite significant challenges, the 12 TWV community members who were interviewed reported the ability to make positive changes within their community, which are expected to help prevent suicide.

## 4 Discussion

In terms of suicide prevention, those living in Guam face unique needs that arise from a range of socio-ecological factors. Due to limited resources in Guam and persistent barriers to increasing the local mental health workforce, community-based approaches, including culturally tailored suicide prevention efforts that address both individual factors and cultural characteristics (23) are essential to help meet the needs of Guam’s Veterans. Our findings provide initial insights regarding unique cultural considerations for suicide prevention among members of this community. Addressing Guam’s elevated suicide rates will require consideration of the complex identities that Guamanian Veterans experience, drawing on strength and resilience, while addressing stigma and barriers to help-seeking. Stigma related to seeking help may be influenced by military culture (34) and Guamanians’ historical identity of being “warriors” and “fighters”.

Further, membership in community and/or religious organizations may complicate individuals’ beliefs and behaviors regarding suicide. Roman Catholicism is traditionally associated with religious opposition to suicide (35), yet the influence of religion on suicide-related beliefs and behaviors is complex and can vary by individual, group, and country characteristics (36). Nonetheless, those interviewed noted shared community awareness that suicide is a problem and conveyed hope for being able to make a difference in preventing Veteran suicide through TWV. Valuing interconnectedness and a shared sense regarding the community’s ability to facilitate change seemed to be important in terms of individuals’ reported progress toward meeting TWV program outcomes.

A vital component of community-based suicide prevention is identification of those at increased risk for suicide and referral to appropriate levels of care. Moreover, lack of access to care is a known risk factor for suicide (37, 38). Therefore, challenges related to accessing healthcare noted by interviewees were concerning. In terms of VA resources, there is a Community Based Outpatient Clinic on the island; however, the nearest Veterans Administration Medical Center (VAMC) is in Honolulu, Hawaii. For Guamanian Veterans in need of the higher level of care offered at a VAMC, the trip to Honolulu requires approximately 8 h of air travel. Further, continuity of care, including longer term relationships with staff, and working with culturally aware and integrated staff are essential to mental health care (39–41).

To meet the growing demand for mental health services in Guam, increased access to resources (e.g., providers) is needed. However, work by Watanabe-Galloway et al. (42) suggests that providers who have limited experience living in Guam are less likely to be culturally aware of the unique population they serve and are unlikely to stay in the community. As such, novel strategies to train Guamanians in mental health fields or incentivize Guamanians to return home after training in other parts of the world should be explored. One such program, enacted with the passage of the 2022 Biråda Act, offers tuition support to Guamanian graduates of programs leading to health profession degrees who agree to return to Guam as part of a service commitment (43, 44).

Additionally, since the pandemic, VA has made an even greater investment in both telehealth and regionally-available suicide prevention services (45). For example, via the Suicide Prevention 2.0 program, VA providers can refer Veterans for specialized suicide prevention services, including Advanced Safety Planning and Cognitive Behavioral Therapy for Suicide Prevention (19, 46). Nonetheless, many of the individuals providing such services are on the mainland, and internet service is necessary. Continued efforts are needed to overcome such barriers.

Like all studies, the present study has limitations. Data were obtained from a small group of community members, resulting in possible selection bias. Findings are not expected to be generalizable; however, results may be transferable to other community-based suicide prevention efforts (47). Further, interviewers, analysts, and co-authors are not part of the Guam community and could have missed culturally relevant statements and contexts. Finally, due to limited variability of responses on both the CMS and CES, interpretation of these measures is limited. We are also unable to compare our data to other communities because there is a lack of established norms or a categorization scheme for such comparisons.

There are areas of import to explore that were not necessarily the focus of the interview questions. Religion plays a strong role on the island. Catholicism is the majority religious affiliation in Guam (48). Although religious faith is a well-established protective factor for suicide (49), this can vary by race, ethnicity, and gender (36, 50). Future research could further assess the role of religion in suicide risk and prevention in Guam.

The present study provides important insights into the unique mental health and suicide prevention needs of Veterans on Guam, as well as initial feedback regarding implementation of TWV. Additional efforts are needed to foster cultural understanding between the rest of the US and Guam to address intrapersonal, social, and chronosystem-related factors. At the same time, qualitative and quantitative findings support the assertion that, even when significant barriers are identified, belief in community capacity coupled with structured community-based strategies result in the individual perception that change is possible.

## Data Availability

The datasets presented in this article are not readily available because the Veterans Health Administration has rules about data security that aim to protect the privacy of Veteran health information. Thus, the data used for this study cannot be made publicly available. Requests to access the datasets should be directed to lisa.brenner@va.gov.

## References

[1] US Department of Health and Human Services. *III.B. Overview of the state - Guam - 2021 [WWW document]*. Maternal and Child Health Bureau. (2021). Available online at: https://mchb.tvisdata.hrsa.gov/Narratives/Overview/08312f4f-d1b8-4988-a861-b6aa9678a13e (Accessed October 7, 2023).

[2] US Census Bureau. (2022). *DECIA Guam demographic profile [WWW document]*. Available online at: https://data.census.gov/table/DECENNIALDPGU2020.DP1?g=040XX00US66&d=DECIA+Guam+Demographic+Profile (Accessed August 11, 2023).

[3] Perez MP. Colonialism, Americanization, and indigenous identity: a research note on Chamorro identity in Guam. Sociol Spectr. (2005) 25:571–91. doi: 10.1080/02732170500176138

[4] Diaz MED. The geopolitical context of Chamorro cultural preservation in Guam, U.S.a. Ethnic Stud Rev. (2012) 35:101–20. doi: 10.1525/esr.2012.35.1.101

[5] David AM. *Guam state epidemiological outcomes workgroup, suicide in Guam, 2021*. Guam Behavioral Health and Wellness Center. (2022).

[6] Ehlman DC Yard E Stone DM Jones CM Mack KA. Changes in suicide rates - United States, 2019 and 2020. Morb Mortal Wkly Rep. (2022) 71:306–12. doi: 10.15585/mmwr.mm7108a5, PMID: 35202357

[7] Monteith LL Holliday R Iglesias CD Sherrill A Brenner LA Hoffmire CA. Suicide risk and prevention in Guam: clinical and research considerations and a call to action. Asian J Psychiatr. (2023) 83:546. doi: 10.1016/j.ajp.2023.103546, PMID: 36958139

[8] US Navy. *History [WWW document]. Naval Base Guam*. (2023). Available online at: https://jrm.cnic.navy.mil/Installations/NAVBASE-Guam/About/History/ (Accessed October 7, 2023).

[9] US Census Bureau. *2020 island areas censuses data on demographic, social, economic and housing characteristics now available for Guam (press release no. CB22-CN.21)*. (2020).

[10] US Department of Veterans Affairs. *State/territories summary reports - Guam [WWW document]*. National Center for Veterans Analysis and Statistics. (2017). Available online at: https://www.va.gov/vetdata/stateSummaries.asp (Accessed June 7, 2023).

[11] Cagurangan MV. *Military size on Guam quietly grows ahead of the marines’ influx [WWW document]. Pacific Island times*. (2022). Available online at: https://www.pacificislandtimes.com/post/military-size-on-guam-quietly-grows-ahead-of-the-marines-influx (Accessed June 7, 2023).

[12] Lendon B. *Us marines officially opens first new base in 70 years on island of Guam [www document]*. CNN. (2023). Available online at: https://www.cnn.com/2023/01/27/asia/new-us-marine-corps-base-guam-intl-hnk-ml/index.html (Accessed November 7, 2023).

[13] US Department of Veterans Affairs. *State summaries Guam [WWW Document]*. Open Data. (2019). Available online at: https://www.data.va.gov/stories/s/State-Summaries_Guam/jmm5-cgfz/ (Accessed February 10, 2023).

[14] US Department of Veterans Affairs. *Veteran population, population tables - the nation: Race/ethnicity, national center for veterans analysis and statistics [WWW document]*. National Center for Veterans Analysis and Statistics. (2022). Available online at: https://www.va.gov/vetdata/veteran_population.asp (Accessed November 7, 2023).

[15] US Department of Veterans Affairs. *Veteran suicide data and reporting (National Veteran Suicide Prevention Annual Report: Data appendix)*. Office of Mental Health and Suicide Prevention. (2022).

[16] Monteith LL Kittel JA Schneider AL Miller CN Gaeddert LA Holliday R . Suicide among Asian American, native Hawaiian, and Pacific islander veterans: rates and methods, 2005-2019. Am J Prev Med. (2023) 66:243–51. doi: 10.1016/j.amepre.2023.09.006 37703953

[17] Monteith LL Kittel J Miller C Schneider AL Holliday R Gaeddert LA . Identifying U.S. regions with the highest suicide rates and examining differences in suicide methods among Asian American, native Hawaiian, and Pacific islander veterans. Asian J Psychiatr. (2023) 89:103797. doi: 10.1016/j.ajp.2023.103797, PMID: 37847965

[18] US Department of Veterans Affairs. *National strategy for preventing veteran suicide 2018–2028*. Office of Mental Health and Suicide Prevention (2018).

[19] Carroll D Kearney LK Miller MA. Addressing suicide in the veteran population: engaging a public health approach. Front Psych. (2020) 11:69. doi: 10.3389/fpsyt.2020.569069, PMID: 33329108 PMC7719675

[20] US Department of Veterans Affairs. *Together with veterans (TWV) [WWW document]. MIRECC / CoE*. (2023). Available online at: https://www.mirecc.va.gov/visn19/togetherwithveterans/ (Accessed September 20, 2023).

[21] Kreisel CJ Wilson LK Schneider AL Mohatt NV Spark TL. Reducing rural veteran suicides: navigating geospatial and community contexts for scaling up a national veterans affairs program. Suicide Life Threat Behav. (2021) 51:344–51. doi: 10.1111/sltb.12710, PMID: 33876499 PMC8252578

[22] Monteith LL Wendleton L Bahraini NH Matarazzo BB Brimner G Mohatt NV. Together with veterans: VA national strategy alignment and lessons learned from community-based suicide prevention for rural veterans. Suicide Life Threat Behav. (2020) 50:588–600. doi: 10.1111/sltb.12613, PMID: 31950557

[23] Garney W Wilson K Ajayi KV Panjwani S Love SM Flores S . Social-ecological barriers to access to healthcare for adolescents: a scoping review. Int J Environ Res Public Health. (2021) 18:4138. doi: 10.3390/ijerph18084138, PMID: 33919813 PMC8070789

[24] McLeroy KR Bibeau D Steckler A Glanz K. An ecological perspective on health promotion programs. Health Educ Q. (1988) 15:351–77. doi: 10.1177/109019818801500401, PMID: 3068205

[25] Tyokighir D Hervey AM Schunn C Clifford D Ahlers-Schmidt CR. Qualitative assessment of access to perinatal mental health care: a social-ecological framework of barriers. Kans J Med. (2022) 15:48–54. doi: 10.17161/kjm.vol15.15853, PMID: 35371389 PMC8942588

[26] Harris PA Taylor R Thielke R Payne J Gonzalez N Conde JG. Research electronic data capture (REDCap)--a metadata-driven methodology and workflow process for providing translational research informatics support. J Biomed Inform. (2009) 42:377–81. doi: 10.1016/j.jbi.2008.08.010, PMID: 18929686 PMC2700030

[27] Hobfoll SE Schröder KEE Wells M Malek M. Communal versus individualistic construction of sense of mastery in facing life challenges. J Soc Clin Psychol. (2002) 21:362–99. doi: 10.1521/jscp.21.4.362.22596

[28] Carroll JM Rosson MB Zhou J. *Collective efficacy as a measure of community*. In: Proceedings of the SIGCHI Conference on Human Factors in Computing Systems, pp. 1–10. (2005).

[29] Creswell JW Clark VLP. Designing and conducting mixed methods research. 3rd ed. Thousand Oaks, CA: SAGE Publications (2017).

[30] Beebe J. Rapid assessment process: An introduction. Walnut Creek, CA: AltaMira Press (2001).

[31] Lewinski AA Crowley MJ Miller C Bosworth HB Jackson GL Steinhauser K . Applied rapid qualitative analysis to develop a contextually appropriate intervention and increase the likelihood of uptake. Med Care. (2021) 59:S242–51. doi: 10.1097/MLR.0000000000001553, PMID: 33976073 PMC8132894

[32] Moon MD. Triangulation: a method to increase validity, reliability, and legitimation in clinical research. J Emerg Nurs. (2019) 45:103–5. doi: 10.1016/j.jen.2018.11.004, PMID: 30616761

[33] Averill JB. Matrix analysis as a complementary analytic strategy in qualitative inquiry. Qual Health Res. (2002) 12:855–66. doi: 10.1177/104973230201200611, PMID: 12109729

[34] Hernandez SHA Morgan BJ Parshall MB. A concept analysis of stigma perceived by military service members who seek mental health services. Nurs Forum. (2017) 52:188–95. doi: 10.1111/nuf.12187, PMID: 27958653

[35] Barry R. The development of the Roman Catholic teachings on suicide. Notre Dame J L Ethics Pub Pol'y. (1995) 9:449. 11653002

[36] Gearing RE Alonzo D. Religion and suicide: new findings. J Relig Health. (2018) 57:2478–99. doi: 10.1007/s10943-018-0629-8, PMID: 29736876

[37] Tadmon D Bearman PS. Differential spatial-social accessibility to mental health care and suicide. Proc Natl Acad Sci. (2023) 120:e2301304120. doi: 10.1073/pnas.2301304120, PMID: 37126686 PMC10175830

[38] Tondo L Albert MJ Baldessarini RJ. Suicide rates in relation to health care access in the United States: an ecological study. J Clin Psychiatry. (2006) 67:517–23. doi: 10.4088/jcp.v67n0402, PMID: 16669716

[39] McGregor B Belton A Henry TL Wrenn G Holden KB. Improving behavioral health equity through cultural competence training of health care providers. Ethn Dis. (2019) 29:359–64. doi: 10.18865/ed.29.S2.359, PMID: 31308606 PMC6604769

[40] Waibel S Henao D Aller M-B Vargas I Vázquez M-L. What do we know about patients’ perceptions of continuity of care? A meta-synthesis of qualitative studies. Int J Qual Health Care. (2012) 24:39–48. doi: 10.1093/intqhc/mzr068, PMID: 22146566

[41] Weaver N Coffey M Hewitt J. Concepts, models and measurement of continuity of care in mental health services: a systematic appraisal of the literature. J Psychiatry Ment Health Nurs. (2017) 24:431–50. doi: 10.1111/jpm.12387, PMID: 28319308

[42] Watanabe-Galloway S Madison L Watkins KL Nguyen AT Chen L-W. Recruitment and retention of mental health care providers in rural Nebraska: perceptions of providers and administrators. Rural Remote Health. (2015) 15:3392. Available at: https://www.rrh.org.au/26567807

[43] Guam Legislature Archives. *36th Guam legislature [WWW document]*. Public law pp. 36–88. (2023). Available online at: https://guamlegislature.com/36th_Guam_Legislature/36th_public_laws_content.htm (Accessed February 10, 2023).

[44] Western Interstate Commission of Higher Education. *Guam community college opens WICHE PSEP office [WWW document]. Resources*. (2023). Available online at: https://www.wiche.edu/resources/guam-community-college-opens-wiche-psep-office/ (Accessed February 10, 2023).

[45] Heyworth L Kirsh S Zulman D Ferguson J Kizer K. Expanding access through virtual care: the VA’S early experience with Covid-19. N Engl J Med. (2020) 1:11. doi: 10.1056/CAT.20.0327

[46] Gujral K Van Campen J Jacobs J Kimerling R Blonigen D Zulman DM. Mental health service use, suicide behavior, and emergency department visits among rural US veterans who received video-enabled tablets during the COVID-19 pandemic. JAMA Netw Open. (2022) 5:e226250. doi: 10.1001/jamanetworkopen.2022.6250, PMID: 35385088 PMC8987904

[47] Konradsen H Kirkevold M Olson K. Recognizability: a strategy for assessing external validity and for facilitating knowledge transfer in qualitative research. Adv Nurs Sci. (2013) 36:66–76. doi: 10.1097/ANS.0b013e318290209d 23644272

[48] The Association of Religion Data Archives. *World religion [WWW document]. National regional profiles - Guam*. (2023). Available online at: https://www.thearda.com/world-religion/national-profiles?u=97c# (Accessed December 7, 2023).

[49] Ullman K Landsteiner A Linskens E MacDonald R McKenzie L Murdoch M . *Risk and protective factors across socioecological levels of risk for suicide: An evidence map (no. #09–009)*. Office of Research and Development, Department of Veterans Affairs, Washington, DC. (2021). 34846828

[50] Barranco RE. Suicide, religion, and Latinos: a macrolevel study of U.S. Latino suicide rates. Sociol Q. (2016) 57:256–81. doi: 10.1111/tsq.12110

[51] Bronfenbrenner U. The ecology of human development: Experiments by nature and design. Cambridge, US: Harvard University Press (1979).

