# Supplementary material for: The “tyranny of distance”: community-based veteran suicide prevention in Guam
Source: Front Public Health. 2025 Jul 28;13:1469973. doi: 10.3389/fpubh.2025.1469973 (PMC12336017; doi:10.3389/fpubh.2025.1469973)
Supplement: Supplementary file 1 [file Supplementary_file_1.docx]

**Supplement A1. Semi-Structured Qualitative Interview Questions**

1. Why did you decide to get involved in the TWV program?
   1. What interested you about the TWV Program?
2. Tell me about your role in the TWV program and/or in the community. (examples could include involvement in community organizations, leadership in groups, family connections, etc.)
3. Tell me about your experience(s) living in your community.
   1. What do you think is unique about your community?
   2. What do you think isn’t unique?
4. How comfortable are people in your community talking about mental health and asking for help?
5. What’s it like to be a Veteran in your community?
6. What comes to mind when you think about veteran suicide in your community?
   1. How can veteran suicide be prevented in your community?
   2. Why do you think veteran suicide is an important problem to address in your community?
7. How has the COVID19 pandemic affected the TWV program or has it?
8. How has COVID19 affected rural communities?
9. What are your expectations for the Together with Veterans (TWV) program? What are your concerns, if any?
10. What impact do you hope TWV has in your community?
11. Which aspects or tools from the Together with Veterans program are the most useful for your community? Why?
12. Which aspects or tools from the Together with Veterans program are the least useful for your community? Why?
13. What would you tell people in other rural communities who are thinking about becoming involved with TWV?

a. What lessons have your learned since starting TWV that you wish you would have known earlier?

b. Are there things that came from TWV that you didn’t expect? What positive things? What about negatives?

1. What do you think about keeping the program going?

a. Is there a sustainability plan in development?

b. What challenges and facilitators do you anticipate facing in sustaining TWV?

1. Regarding Veteran suicide prevention, what do you think your community will be like 5 years from now? What challenges and facilitators do you think might impact Veteran suicide prevention?
2. What didn’t I ask that I should know?
