# Supplementary material for: The “tyranny of distance”: community-based veteran suicide prevention in Guam
Source: Front Public Health. 2025 Jul 28;13:1469973. doi: 10.3389/fpubh.2025.1469973 (PMC12336017; doi:10.3389/fpubh.2025.1469973)
Supplement: Supplementary file 2 [file Supplementary_file_2.docx]

**Supplement A2. Together with Veterans Implementation Questionnaire (TWV-IQ) Results**

| **Code for Inclusion in**  **Table 2** | **Communal Mastery Scale (Possible score range 10-40; higher scores indicate higher communal mastery)** | **Mean (std)** | **Median (IQR)** |
| --- | --- | --- | --- |
| - | Communal Mastery Total Score | 32.3 (3.6) | 31.5 (30.0, 34.5) |
| **-** | **Collective Efficacy Scale (Scale: 1=Strongly disagree; 5=Strongly Agree; Possible score range is 5-25; higher scores indicate more beliefs in the capacity of the community to achieve its goals)** | **Mean (std)** | **Median (IQR)** |
| A | Collective Efficacy Scale Mean Score | 21.3 (4.8) | 23.0 (19.0-25.0) |
| **-** | **Opinions about Together with Veterans Program**  **(Scale: 1=Very Dissatisfied; 5=Very Satisfied)** | **Mean (std)** | **Median (IQR)** |
| **B** | **Planning and Implementation (Overall)** | 4.3 (0.6) | 4.0 (4.0, 5.0) |
| B1 | Planning process used to prepare objectives for the initiative | 4.3 (0.6) | 4.0 (4.0, 5.0) |
| B2 | Follow-through on the initiative's activities | 4.2 (0.8) | 4.0 (3.5, 5.0) |
| B3 | Strength and competence of staff | 4.4 (0.7) | 4.5 (4.0, 5.0) |
| **C** | **Leadership (Overall)** | 4.2 (0.6) | 4.3 (3.8, 4.5) |
| C1 | Clarity of the vision for where the initiative should be going | 4.5 (0.5) | 4.5 (4.0, 5.0) |
| C2 | Strength and competence of leadership | 4.3 (0.7) | 4.0 (4.0, 5.0) |
| C3 | Sensitivity to and awareness of cultural issues | 4.4 (0.7) | 4.5 (4.0, 5.0) |
| C4 | Use of the media to promote awareness of the initiative's goals, actions, and accomplishments | 3.5 (0.8) | 3.0 (3.0, 4.0) |
| C5 | Opportunities for members of the initiative to take leadership roles | 4.2 (0.7) | 4.0 (4.0, 5.0) |
| **D** | **Community Involvement in the Coalition (Overall)** | 3.8 (0.8) | 3.8 (3.1, 4.6) |
| D1 | Participation of influential people from key sectors of the community | 3.7 (1.0) | 4.0 (3.0, 4.0) |
| D2 | Participation of community members | 3.8 (0.9) | 3.5 (3.0, 4.5) |
| D3 | Diversity of members in the initiative | 4.1 (0.7) | 4.0 (4.0, 4.5) |
| D4 | Involvement of respected leaders in the community | 3.5 (1.2) | 3.5 (2.5, 4.5) |
| D5 | Involvement of grassroots organizations (e.g., non-profits) | 4.2 (0.8) | 4.0 (3.5, 5.0) |
| D6 | Involvement of civic organizations (e.g., local government organizations) | 3.9 (0.9) | 4.0 (3.0, 5.0) |
| **E** | **Progress and Outcome (Overall)** | 4.1 (0.6) | 4.0 (3.9, 4.4) |
| E1 | Progress in meeting the initiative's objectives (e.g. strengthening suicide prevention efforts among rural Veterans in your community) | 4.0 (0.7) | 4.0 (3.5, 4.5) |
| E2 | Success in generating resources for the initiative | 4.2 (0.6) | 4.0 (4.0, 4.5) |
| E3 | Success in generating community support for the initiative | 4.0 (0.6) | 4.0 (4.0, 4.0) |
| E4 | The initiative's contribution to the goal of preventing suicide among rural Veterans in your community | 4.2 (0.6) | 4.0 (4.0, 4.5) |
| **-** | **Overall Suggestions and Approval Rating** | 4.2 (0.6) | 4.0 (4.0, 4.5) |
| **F** | **Is the community better off today because of this program?** | **N(%)** | |
| - | Yes | 12 (100%) | |
| - | No | 0 | |
| **G** | **I feel confident that these efforts will continue in my community and are sustainable.** | **N(%)** | |
| - | Yes | 12 (100%) | |
| - | No | 0 | |
