# Supplementary material for: The “tyranny of distance”: community-based veteran suicide prevention in Guam
Source: Front Public Health. 2025 Jul 28;13:1469973. doi: 10.3389/fpubh.2025.1469973 (PMC12336017; doi:10.3389/fpubh.2025.1469973)
Supplement: Supplementary file 3 [file Supplementary_file_3.docx]

**Supplemental Table B. Socioecological Levels, Themes, and Quotes**

| **Socioecological Level** | **Theme** | **Quote** |
| --- | --- | --- |
| Intrapersonal - Individual (Community Member) | Identities | “Veterans can be conflicted here with their local identity and their patriotic identity... And so when you are mixing the two, it can be somewhat of an internal conflict where some veterans have that challenge.”  “... you love your country. You served your country. You go to war, and it's such a conflict of values... and then you come back to Guam where the culture is still so strong, and it kind of intensifies the displacement and the self-hatred… and that moral, the sense of the moral injury.”  [On being half Chamorro and of another ethnic background: "Guam is a very passionate place and very family oriented but… it's kind of hard to be integrated into a place that is... only half yours."    “We're a very proud island. We're proud of who we are. We're proud of where we come from. And so when it comes to suicide ideation... having that identity crisis. Am I a Veteran? Am I a local native, indigenous native? Am I a native islander? They have a lot of these internal battles, and culturally speaking, it's when you're having two different ideals competing with each other… then you start to wonder, where do I actually belong?” |
|  | Mental Health Stigma and Resistance to Treatment – Individual Experiences | “Show our feelings, show when we're hurt, and that kind of rolled into the military. We were bred for this. We were bred to fight. Our native people are bred to fight. We're not supposed to cry. We're not allowed to cry. We're not to show our feelings.”  “I think everyone has a little hint of mental health in them, but it's just such a, like it's such a bad word to say, and people look down on you or talk about you behind your back, which creates more anxiety.”  "So I think that definitely, you know that multigenerational trauma goes undetected or untreated then it manifests itself further."    "Help is not a word that any of us use, because when you ask for help that means you're retreating. That means you've already failed your mission."  “A lot of us, every single one of us knows someone that’s killed themselves….I should’ve been there, I could’ve been there, but we can’t blame ourselves because, at the end of the day, it’s—blaming ourselves for not being there for that person...blaming ourselves isn’t gonna get anything done." |
| Social – Interpersonal (community members interacting with each other) | Perceptions of Military/Veterans | “Very patriotic, the community… everyone wants to help the military and veteran community. And no one ever makes you feel uncomfortable… I think because it’s a small community and a lotta folks opt to go in the military because the employment market’s not very robust out here. So it’s like okay, right from high school I’m gonna join the military. So you have a lot of, lot of generations of military service."  “It's just so common everyone’s a Veteran here. Everyone's gonna serve. I can guarantee you go to any house here and say you know a Veteran and you have a Veteran in your family, and I can almost guarantee they’ll only say yes.”  “We're all family here. Everybody knows each other. We're loving. We're compassionate people. We really care. And I keep going back to we're warriors and we're fighters because after all these years, we still haven't rolled on our back.”  “There’s a lot of positive regard for our Veterans and respect.”  "We wanna serve, and that's just in our bloodline. We're warriors, so we serve in the United States.” |
| Social - Community (community member interacting with organization) | Access to Care – Individual Experiences Interacting with Healthcare System | "The price of freedom and can’t even get simple health care. Are we entitled to get health care? Yeah, absolutely. But I have to leave my family again in order to frickin’ go and get my health care, which is just unjust."  “Because they don’t wanna keep going back [for mental health care]. That’s what I hear is they’re like, oh well I’ve been there three or four times and no one’s helped me or I’m still waiting for an appointment. Oh well, forget them, you know, I’ll just deal with it on my own...”  “...we come back, and there's just no service, barely any services here. And I’m not gonna say that we don't have services, but I think there are things within our system that can be a little bit altered so we can get better care or more care."  “I have to fly off island, everything has to be off island. And that, that's what drives most of the Veterans and even just regular civilians off the island is because there's no resources that’s available here.” |
|  | Mental Health Stigma and Difficulty Accessing Resources/ Barriers to Access to Treatment | “Even for the people that aren’t from our local community who are in the military, and we provide aftercare, oftentimes, they wouldn't wanna say, they wouldn't admit it because that may impact their career in the military… When you think about it, just the issue of mental health, I think that's across all cultures, I think. I think that's where it’s not unique. The stigma of mental health and seeking the help.”  “So even in regard to therapy, if we're doing like cognitive behavioral therapy, it's more effective when you understand the Chamorro cultural lens … Because our schemas are very different.”  "If you already have issues you don’t wanna, I mean you go to the Department of Mental Health... there’s a security guard up front.”  “People are somewhat ashamed that someone committed suicide or took their life through suicide. The Catholic Church frowns upon that with the old notions of you're not gonna go to heaven and that kind of thing.” |
| Social - Organizational (organizations interacting with each other) | Reasons for Adoption | “Together with Veterans was this beautiful opportunity for us to try to work with our local nonprofits in a way that we can start sharing our knowledge with each other in order to build our own strengths.”  "I’ve had several soldiers in the past who’ve committed suicide, that I know, and it’s one of those things that you think you can catch, or you know they won’t do it, but it happens to those people and you’re just like, wow, I wanna be a part of that organization that can help in any way, or just reach out, or just be an ear."  “To be the driving force. To help us here on Guam raise awareness for suicide among its Veterans.” |
|  | Hope for Raising Awareness | “What I see with the TWV program is a collaborate effort. What we identify with a lot of the community partners is each agency, organization, whatever it may be, they have their mission. They have their goals, and we’ve identified that a lot of these things are kept in silos...But Together With Veterans, I think, is a great opportunity to get everybody in the same room, on the same page, in the same chatroom, for cryin' out loud."  "I think the Together with Veterans is on the right track. ...they’re collaborating with the different organizations or trying to gather the data and information that’s necessary to see how would you go forward. I’ve never seen anything like this before so it’s really new."    "...it’s just getting that message out. Getting folks trained up. Getting people engaged and wanting to participate. And knowing that if, when they do participate, there’s a reward at the end."    "TWV is the closest thing we’ve got going on for mass awareness."  “We’re setting the foot work or the groundwork for better and more care for the Veterans. So the younger ones that are in right now and getting out are, you know, have been, that are here, but don't even seek any better Veteran care.... they finally take on, you know, they'll have that help, they'll have those resources.”    “I think TWV is helping create relationships which helps give purpose and give someone hope that there are people out there for them.”  "And then the TWV folks that, you know kinda have the safe talk training and okay, they’re recognizing the signs that yeah this person might be, you know, teetering on the edge of something. Let’s get him plugged into some mental health services. So I think, I think it’s, TWV would be that interface." |
|  | Barriers and Facilitators | “I think it helps that TWV is so flexible in how they allow us to utilize the funding. … It's not a typical VA or even federal program where there's so much red tape that you don't even want their money… There's a good trust in that we know our community and what's gonna draw people.”  "Honestly I feel the VA, the Vet Center in particular can fund projects a lot quicker than they do instead of putting barriers up for us…. I think the barriers are slowing us down more than they are helping us to develop the program." |
| Social - Public Policy and Federal Systems (national systems of care) | Part of, yet Isolated from, the Mainland U.S. | “Way out in the middle of nowhere. We’re not the United States. But we are the United States.”  “I mean I know we’re all American, but just like the cultural aspect when it comes to maybe interpreting things or perceiving things, it may be a little bit tough for people to understand.”    “Guam has that small town American feel...We’re the tip of the spear, we’re gonna be the first line of defense against other countries ...And then the community comes second.”    "You know that tyranny of distance you always hear. You know it’s like Guam is so far away from everything and, and you know. I’m going stateside and folks are asking, what type of currency we use? And like, the U.S. dollar.”    “I can tell you that the Veteran community here on Guam feels like the red-headed stepchild. Maybe being so far away from easy access to care, whether it's healthcare or on the benefits side.”    “And it's very segregated from the Western systems and treatment interventions, things like that.” |
|  | Access to Care – Federal Systems | “Guam politically, we’ve been a colony specifically of America since after the Spanish-American War…for a long time, we’ve been governed by just the US military as wards, like wards of the state.”  “Because there's so much distrust. Right? Anything related to the VA, it's like you don't really know how it's gonna end up.”  “We don't have a large kind of provider pool in the islands. Geographics is a huge setback. Because to get any kind of residential treatment...that's an eight-hour flight. And then they're disconnected from their families, which have been their protective factors naturally.”  "I believe it [facilitation/transition of care from doctor to doctor] has to come from a Veteran organization because it’s a lot easier to talk to another Vet when they know you’re a Vet. So, like say, for example, if it’s a civilian doctor, right, but you have that liaison or that mediator, whatever it is, in the middle that’s a Vet, it makes the transition so much easier because sometimes Vets will not open to a civilian."    "I’m actually not very proud of the type of services that we have at this point because other parts of the country that's just not the case. It’s much more responsive, much more enriched, and much more well-funded."  “We need an actual place for us [veterans] because we can’t be treated like everybody else. We should as human beings, but what we’ve dealt with is not the same as everybody else and I think we should have a specific place for us to go and get the help that we need.”  “Also broken promises from the federal side. So, I believe in the past, visits from the [VA Leadership] … Veterans had a town hall meeting or some type of roundtable discussion to voice their concerns. And yeah, yeah, we'll look into it, and then there's no follow-up.” |
| Chronosystem (change over time) | Geopolitical and Colonial History | “We have the most Veterans per capita, I’m just saying, I mean I don’t know if it's changed, but even, even as far as enlisting per capita we’re the highest. We’re ranked pretty high on the, you know, we’re tip of the spear, we’re [a] strategic place for military.”  “...when you take a look at Guam’s history, Catholicism is very ingrained into the culture.”  “Well, there’s an old saying I say and it’s been true for my family members, equal in war, but not equal in peace. As you might know, as a VA and I'll say it, we’re the lowest or second lowest funded VA in the country. And, you know, we have one of the highest enlistment rates in the country. So that's such an oxymoron, such a paradox. So yeah I think a lot of Veterans here are very frustrated and angry.” |
